# PRECEPT: an evidence assessment framework for infectious disease epidemiology, prevention and control

**DOI:** 10.2807/1560-7917.ES.2017.22.40.16-00620

**Published:** 2017-10-05

**Authors:** Thomas Harder, Anja Takla, Tim Eckmanns, Simon Ellis, Frode Forland, Roberta James, Joerg J Meerpohl, Antony Morgan, Eva Rehfuess, Holger Schünemann, Teun Zuiderent-Jerak, Helena de Carvalho Gomes, Ole Wichmann

**Affiliations:** 1 Robert Koch Institute (RKI), Berlin, Germany; 2 National Institute for Health and Care Excellence (NICE), London, United Kingdom; 3 Norwegian Institute of Public Health, Oslo, Norway; 4 Scottish Intercollegiate Guidelines Network (SIGN), Edinburgh, United Kingdom; 5 Cochrane Germany, Medical Center – University of Freiburg, Freiburg, Germany; 6 Glasgow Caledonian University, Glasgow, United Kingdom; 7 Institute of Medical Informatics, Biometry and Epidemiology, University of Munich, Munich, Germany; 8 Department of Health Research Methods, Evidence, and Impact, McMaster University Health Sciences Centre, Hamilton, Canada; 9 Department of Thematic Studies -Technology and Social Change, Linköping University, Linköping, Sweden; 10 European Centre for Disease Prevention and Control (ECDC), Stockholm, Sweden

**Keywords:** evidence-based medicine, methodology, systematic reviews, GRADE, risk of bias, decision-making, meta-analysis

## Abstract

Decisions in public health should be based on the best available evidence, reviewed and appraised using a rigorous and transparent methodology. The Project on a Framework for Rating Evidence in Public Health (PRECEPT) defined a methodology for evaluating and grading evidence in infectious disease epidemiology, prevention and control that takes different domains and question types into consideration. The methodology rates evidence in four domains: disease burden, risk factors, diagnostics and intervention. The framework guiding it has four steps going from overarching questions to an evidence statement. In step 1, approaches for identifying relevant key areas and developing specific questions to guide systematic evidence searches are described. In step 2, methodological guidance for conducting systematic reviews is provided; 15 study quality appraisal tools are proposed and an algorithm is given for matching a given study design with a tool. In step 3, a standardised evidence-grading scheme using the Grading of Recommendations Assessment, Development and Evaluation Working Group (GRADE) methodology is provided, whereby findings are documented in evidence profiles. Step 4 consists of preparing a narrative evidence summary. Users of this framework should be able to evaluate and grade scientific evidence from the four domains in a transparent and reproducible way.

## Introduction

The potential fallacies of relying solely on expert opinion to establish best practice in clinical decision-making and public health policies are well known globally [1]. In guideline development, it is standard practice to draw on systematic reviews of the available evidence. For evidence of benefits and harms, well conducted randomised controlled trials (RCTs) minimise bias and systematic reviews of these are commonly used in decision-making. However, observational studies are important for answering public health questions, not least because in many cases they are the only available or feasible source of empirical evidence [2].

Judging the effectiveness of infectious disease prevention and control interventions creates challenges related to the population-level effects and long-term aspects of the intervention. In addition, a variety of other elements need to be considered in decision-making, including disease burden, risk factors for infection or mode of transmission. In adopting the concept of ‘best available evidence’ [3], evaluating the benefits and harms along the full causal chain from intervention to outcomes within a given context requires a variety of fit-for-purpose methods from multiple disciplines.

Although considerable progress has been made regarding the use of systematic reviews for public health decision-making, the approaches currently used for conducting and appraising systematic reviews in public health have their limits. They regularly report effect estimates and risk of bias, but often do not assess the certainty of the evidence for the entire body of such across outcomes. Moreover, traditional approaches mainly focus on intervention effectiveness and safety, but do not provide a generalised approach that addresses all factors relevant to decision-making in infectious disease prevention and control, such as the epidemiology, interventions as well as diagnostics and risk factors.

New evidence appraisal and grading system approaches that incorporate information from studies with different designs have been developed. The most prominent system, developed by the Grading of Recommendations Assessment, Development and Evaluation Working Group (GRADE) [4], has been widely applied in clinical medicine as well as public health. A working group established by the European Centre for Disease Prevention and Control (ECDC) examined the application of GRADE to infectious disease prevention and control [5] and initiated the Project on a Framework for Rating Evidence in Public Health (PRECEPT). The PRECEPT consortium was established in 2012 with representatives from European public health agencies, academic institutions and ECDC. The first results of this project were published earlier [6,7].

An approach to infectious disease prevention and control that adheres to the principles of evidence-based public health, using a defined framework for the assessment of the certainty in the evidence, has a number of advantages over conventional approaches. In particular, such an approach:

- Helps to improve the quality of the resulting public health recommendations.
- Reduces anticipated or actual arbitrary decisions.
- Improves transparency.
- Builds trust and supports the acceptance of recommendations by professionals and the public.
- Helps to compare recommendations endorsed by different countries or institutions.

The approach presented here applies the most advanced methodology for assessing certainty of the evidence, the GRADE methodology. Even though the individual elements (e.g. the GRADE methodology, PICO question framing and systematic review) of the proposed approach are not new, they have never been integrated into one comprehensive framework that guides users from identification of the relevant research questions to preparation of a final evidence assessment summary for the area of infectious disease prevention and control. Moreover, in contrast to other approaches, the framework puts particular emphasis on question framing and the selection of appropriate risk of bias tools. The framework provides evidence assessment guidance in infectious disease epidemiology, prevention and control, but is not designed to conduct a rapid assessment for the purpose of answering urgent questions in public health crises or emergencies.

PRECEPT intends to provide methodological guidance for public health agencies, scientists working in the field of evidence-based public health, and other institutions and individuals involved in appraising evidence and developing public health guidance with a focus on infectious diseases. Here we present an overview of the PRECEPT approach (see also Supplementary Material [8]).

## Domains used in the PRECEPT framework

The PRECEPT framework focuses on the following four domains:

- Disease burden (significance of the problem), which encompasses studies on the incidence, prevalence and severity of diseases and complications, as well as studies on the perception of diseases in target populations. For example, what is the incidence of hepatitis B in sex workers in eastern European countries?
- Infection and disease risk factors (causes of the problem), which encompasses studies on preventable and non-preventable risk factors for infection, disease and complications. For example, is sepsis acquired in the neonatal intensive care unit a risk factor for cerebral palsy?
- Diagnostics (detection of the problem), which encompasses studies on diagnostic accuracy (sensitivity and/or specificity of diagnostic tests/measures). For example, what is the sensitivity and specificity of tests for tuberculosis in children?
- Interventions (consequences of action against the problem), which encompasses studies examining efficacy, effectiveness and adverse effects. For example, what is the effectiveness of vaccination of infants against rotavirus for the prevention of hospitalisation?

## Four-steps to assessing evidence

**Figure 1:**
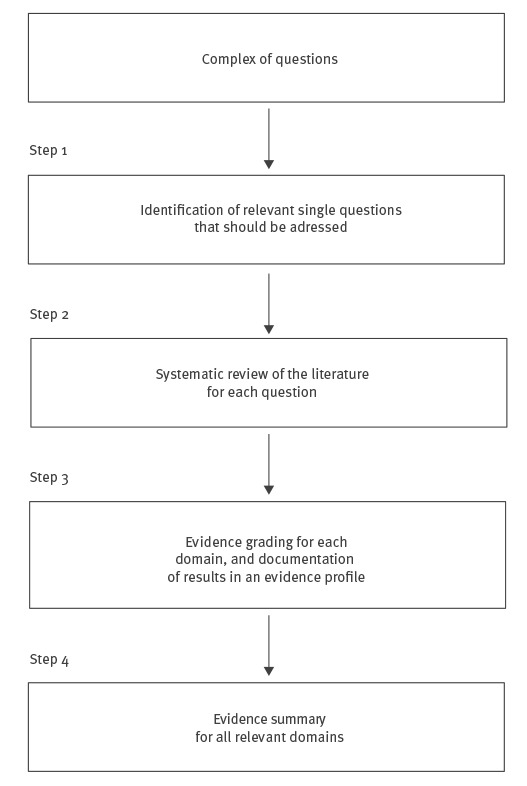
Flowchart for working with the PRECEPT methodology

The general approach to this process of assessing evidence within these four domains consists of four steps (Figure 1).

### Step 1: Identify the relevant questions

PRECEPT proposes applying the extended Patient/Population, Intervention, Comparator/Comparison/Control, and Outcome (PICO) format and integrating other questions to the extent possible.

PICO is considered to be the most appropriate way of framing questions related to interventions (domain (iv)) [2,9]. However, for studies on disease burden (domain (i)), it can be changed to focus on population, condition (i.e. disease) and context [10]. For risk factors (domain (ii)), the PICO format can be easily modified by replacing ‘intervention’ with ‘exposure’ and ‘comparator’ with ‘absence of risk factor’. Furthermore, different risk factors can be compared with each other (risk factor x vs risk factor y) and different risk factor levels to establish an exposure–response relationship. For diagnostic accuracy studies (domain (iii)), PICO can be interpreted as population, index test, comparator test and outcome (target condition) [11]. Examples of PICO questions for all domains are shown in Table 1.

**Table 1:** Application of PICO to four infectious disease domains, disease burden, risk factors, diagnostics and intervention

PICO element	Suggested adaption/addition according to domain	Example
**Domain i: Disease burden**		
Population	No adaptation necessary	Sex workers
No existing element	Condition	Hepatitis B
	Context	Countries in Eastern Europe
PICO question: What is the incidence of hepatitis B in sex workers in Eastern European countries?		
**Domain ii: Risk factors**		
Population	No adaptation necessary	Hospitalised patients
Intervention	Exposure or risk factor	Prior antibiotic use
Comparator	Absence of exposure or risk factor	No prior antibiotic use
Outcome	No adaptation necessary	Infection with carbapenemase-producing *Enterobacteriaceae*
PICO question: In hospitalised patients, does prior antibiotic use, compared with no prior antibiotic use, pose a risk of carbapenemase-producing *Enterobacteriaceae* infection?		
**Domain iii: Diagnostics**		
Population	No adaptation necessary	Children < 5 years of age
Intervention	Index test	Interferon gamma release assays
Comparator	Comparator test	Tuberculin skin test
Outcome	No adaptation necessary	Tuberculosis
PICO question: What is the sensitivity and specificity of interferon gamma release assays compared with the tuberculin skin test for tuberculosis in children < 5 years of age?		
**Domain iv: Intervention**		
Population	No adaptation necessary	Children < 5 years of age
Intervention		Infant rotavirus vaccination
Comparator		No vaccination
Outcome		Diarrhoea
PICO question: In children < 5 years of age, does infant rotavirus vaccination, compared with no vaccination, prevent diarrhoea?		

PICO: population, intervention, comparator, outcomes.

In infectious disease prevention and control, researchers are regularly confronted with complex of questions that have to be addressed in combination. For example, the question, ‘Should vaccination against rotavirus be recommended for all infants?’ comprises multiple questions from different fields that need to be addressed e.g.:

- What is the incidence of rotavirus infection among children < 5 years of age?
- Is age a risk factor for rotavirus infection among children < 5 years of age?
- What is the effectiveness of vaccination against rotavirus?
- What is the risk of intussusception associated with the vaccine?

In such situations, developing a logic model (conceptual diagram) tends to be helpful [12] to identify and prioritise all relevant questions, and to place these in context. In systematic reviews, a logic model is a graphical representation that helps in scoping the review, defining and conducting the review, and making results from the review relevant to policy and practice [13].

### Step 2: Perform the systematic review

**Figure 2:**
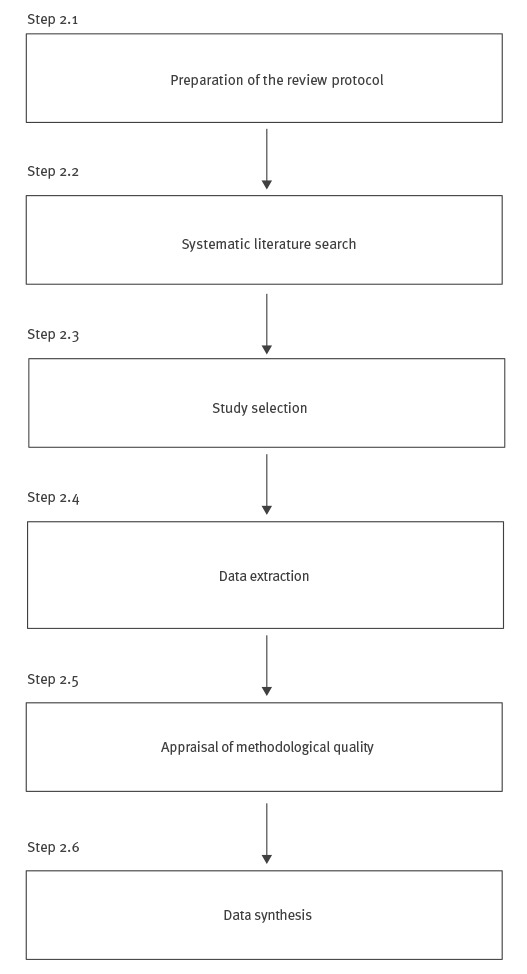
Overview of the systematic review process

Evidence should be identified and synthesised using a rigorous systematic review process. A systematic review usually includes six steps (Step 2.1. to 2.6. as parts of step 2 of the PRECEPT workflow, see Figure 2).

For more extensive information on conducting a systematic review, readers are referred to the literature [9,14].

#### Assessment of methodological quality (risk of bias)

**Figure 3:**
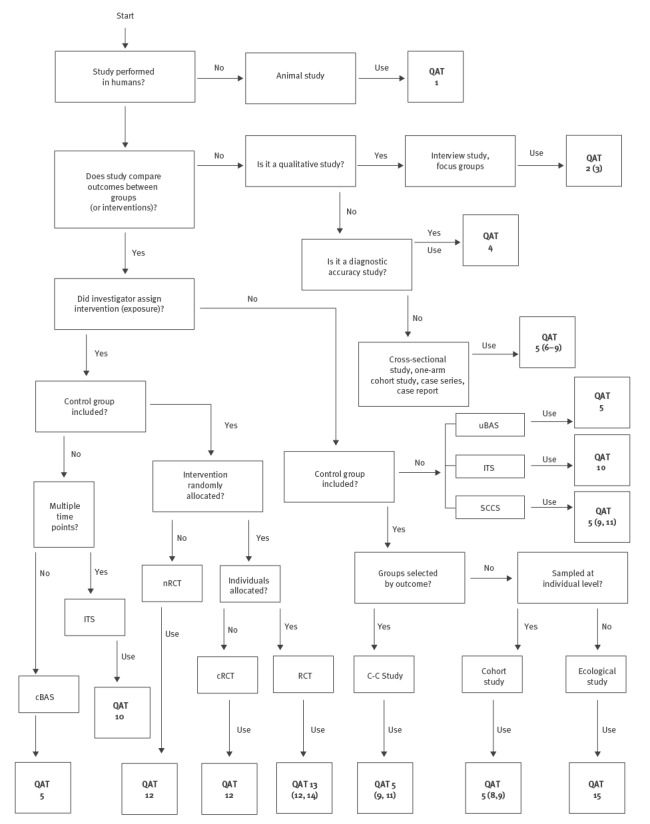
PRECEPT algorithm for identifying quality appraisal tools according to study design

For appraising the methodological quality (risk of bias) of each study identified during the systematic review, the PRECEPT framework proposes using specific quality appraisal tools (QATs) according to study design [6]. A selection of 15 QATs, identified and selected during the first phase of the project by applying a systematic review-based approach [6], are proposed (Figure 3, Table 2). For each study design, the algorithm leads to the identification of a single QAT or a group of QATs. Risk of bias should be assessed in the form of a judgment rather than a score. It is suggested that the Cochrane classification scheme for bias is used: (i) high risk of bias; (ii) low risk of bias, and (iii) unclear risk of bias [14].

cBAS: controlled before–after study; C–C study: case–control study; cRCT: cluster-randomised controlled trial; ITS: interrupted time series; nRCT: non-randomised controlled trial; QAT: quality appraisal tool; RCT: randomised controlled trial; SCCS: self-controlled case series; uBAS: uncontrolled before-after study.

^a^ QATs mentioned first are recommended as first choice, whereas those in parentheses can be used alternatively.

^b^ QAT numbers refer to Table 2.

**Table 2:** PRECEPT-recommended quality appraisal tools for assessing risk of bias according to study design^1^

QAT	Reference	Animal study	Before–after study (controlled)	Before–after study (uncontrolled)	Case–control study	Case report	Case series	Cohort study	Cross-sectional study	Diagnostic accuracy study	Ecological study	Focus groups	Interrupted time series	Interview study	Non-randomised controlled trial	Cluster-randomised controlled trial	Randomised controlled trial	Self-controlled case series
1	SYRCLE [25]	**X**	–	–	–	–	–	–	–	–	–	–	–	–	–	–	–	–
2	NICE (qualitative) [9]	–	–	–	–	–	–	–	–	–	–	**X**	–	**X**	–	–	–	–
3	CASP [26]	–	–	–	–	–	–	–	–	–	–	**X**	–	**X**	–	–	–	–
4	QUADAS-2 [27]	–	–	–	–	–	–	–	–	**X**	–	–	–	–	–	–	–	–
5	Cho [28]	–	**X**	**X**	**X**	**X**	**X**	**X**	**X**	–	–	–	–	–	–	–	–	**X**
6	Hoy [29]	–	–	–	–	–	–	–	**X**	–	–	–	–	–	–	–	–	–
7	Al-Jader [30]	–	–	–	–	–	–	–	**X**	–	–	–	–	–	–	–	–	–
8	SIGN (cohort) [31]	–	–	–	–	–	–	**X**	–	–	–	–	–	–	–	–	–	–
9	NOS [32]	–	–	–	**X**	–	–	**X**	–	–	–	–	–	–	–	–	–	**X**
10	EPOC [33]	–	–	–	–	–	–	–	–	–	–	–	**X**	–	–	–	–	–
11	SIGN (case–control) [31]	–	–	–	**X**	–	–	–	–	–	–	–	–	–	–	–	–	**X**
12	NICE (intervention) [9]	–	–	–	–	–	–	–	–	–	–	–	–	–	**X**	**X**	**X**	–
13	Cochrane [14]	–	–	–	–	–	–	–	–	–	–	–	–	–	–	–	**X**	–
14	SIGN (RCT) [31]	–	–	–	–	–	–	–	–	–	–	–	–	–	–	–	**X**	–
15	NICE (correlation) [9]	–	–	–	–	–	–	–	–	–	**X**	–	––	–	–	–	–	–

–: QAT not applicable to study design; CASP: Critical Appraisal Skills Programme; EPOC: Effective Practice and Organisation of Care; NICE: National Institute for Health and Care Excellence; NOS: Newcastle-Ottawa-Scale; PRECEPT: Project on a Framework for Rating Evidence in Public Health; QUADAS: Quality appraisal of diagnostic accuracy studies; QAT: quality appraisal tool; SIGN: Scottish Intercollegiate Guidelines Network; SYRCLE: Systematic review centre for laboratory animal experimentation. **X**: QAT applicable to study design.

^1^ For non-randomised intervention studies, PRECEPT users might consider the newly developed ROBINS-I tool [34].

#### Synthesis of data

In the case of quantitative data, data synthesis can be conducted using meta-analytic techniques. If statistical pooling appears to be inappropriate, e.g. if interventions are too heterogeneous to be grouped in a meaningful way, if data are highly heterogeneous or if study designs differ considerably, a tabular, graphical or narrative synthesis might be more useful [15].

#### Use of existing systematic reviews

It is estimated that a new full systematic review takes between six to 24 months, but using existing systematic reviews for the development of new evidence assessments can shorten this by one to two thirds [16]. The use of existing systematic reviews might therefore reduce efforts and costs, making the evidence assessment process more efficient. Existing systematic reviews might complement the PRECEPT framework in various ways, e.g. by identifying studies, by answering full questions or by providing search strategies. Before using an existing systematic review, the need for an update should be evaluated. The process of identifying, assessing and applying existing systematic reviews should follow the steps proposed by Robinson et al. [17], and tools such as AMSTAR [18] or ROBIS [19] should be used to assess the methodological quality of existing systematic reviews.

### Step 3: Apply the evidence-grading system and document the results

PRECEPT recommends a standard approach that uses the GRADE methodology to evidence-grading across all four types of domains.

#### Quantitative evidence

According to GRADE, the certainty in the evidence indicates the extent to which one can be confident that the estimate of effect is correct [4]. The units of analysis of GRADE are outcomes, meaning all assessments focus on the outcome of the intervention. At the beginning of the evidence review process, each outcome is rated from 1 to 9 regarding its importance to the decision, where outcomes rated 7 to 9 are regarded as ‘critical’, 4 to 6 as ‘important’, and 1 to 3 as ‘of less importance’.

**Figure 4:**
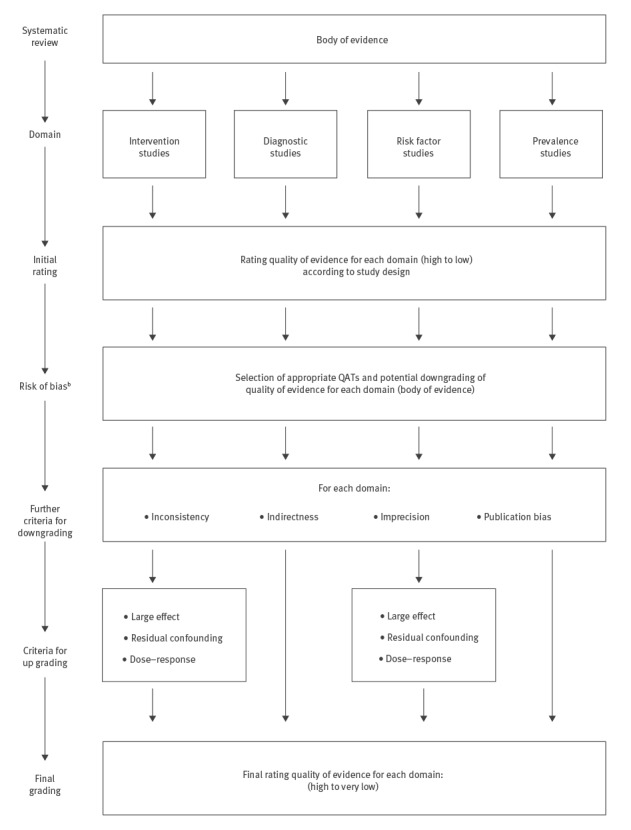
PRECEPT flow chart for grading quantitative evidence certainty according to domain using the GRADE methodology

For questions related to burden of disease (domain (i), outcomes can be measures of incidence or prevalence, as well as mortality or disability-adjusted life years. For questions regarding risk factors (domain (ii)), outcomes are those variables that are dependent on the risk factor. For diagnostics (domain (iii)), true positives, true negatives, false positives and false negatives are regularly used as surrogates for subsequent clinical outcomes. For questions regarding interventions (domain (iv)), outcomes are endpoints of clinical trials or observational studies. Taking the entire body of evidence, not an individual study, on one outcome into account, four levels of certainty in the evidence, i.e. confidence in the estimate of the effect, are applied to the results of the review:  very low, low, moderate and high. For interventions, RCTs are initially graded as high certainty, whereas all types of observational studies are classified as low certainty. Based on a defined set of criteria, decreasing (downgrading) or increasing (upgrading) by one or two levels is possible. Five criteria are applied for downgrading: (i) risk of bias, (ii) inconsistency, (iii) indirectness, (iv) imprecision and (v) publication bias. Three criteria are used to upgrade the certainty in the evidence: (i) large effect, (ii) evidence for a dose-response relationship and (iii) all plausible confounding would have reduced the effect. The lowest quality level among all critical outcomes defines the overall level of evidence across all outcomes. PRECEPT proposes the following unified approach using GRADE for all four domains (Figure 4), which is consistent with the current GRADE approach [4,20-22]:

- For each body of evidence related to an outcome, an initial rating of the certainty in the evidence is performed. For some of the domains, this initial rating depends on study design.
- Risk of bias is assessed using the appropriate QAT for the individual studies (see Step 2). A judgment about the risk of bias is made for the body of evidence, and evidence certainty can be downgraded, if necessary.
- Thereafter, the other GRADE criteria for downgrading the certainty in the evidence (inconsistency, indirectness, imprecision, publication bias) are applied.
- For the domains of ’intervention studies’ and ‘risk factor studies’, upgrading of the certainty in the evidence is possible, according to the criteria introduced by GRADE. Evidence certainty should usually not be up graded after having been downgraded. It is currently unclear whether and how upgrading criteria are applicable to bodies of evidence on prevalence and diagnostics.

GRADE: Grading of Recommendations Assessment, Development and Evaluation Working Group.

#### Qualitative evidence

For rating the evidence certainty of qualitative studies, PRECEPT proposes to assess limitations in the individual studies using the appropriate QATs. In addition, users of PRECEPT might consider the GRADE–CERQual (Confidence in the Evidence from Reviews of Qualitative research) approach for assessing the confidence of evidence from reviews of qualitative research [23].

### Step 4: Prepare an evidence summary

At the end of the evidence appraisal process, a narrative evidence summary for communication of the results should be prepared. The following points should be captured: (i) the overall question, which describes the key question of the review, (ii) detailed questions, which lists the questions addressed by the review, and (iii) the volume of evidence, which describes the studies identified during the review, and (iv) the evidence statement and grading, which summarises the evidence which was identified by the review and the results of the grading process.

## Conclusion

Over the past three years, the PRECEPT team has developed this framework for the assessment of evidence in the field of infectious disease epidemiology, prevention and control. Currently, the application of the framework is being tested in other systematic reviews and projects [16,24]. Furthermore, a consultation process will be carried out to obtain feedback and collect suggestions for improvement. This process might lead to further refinements and adaptations of the framework.
